# Reverse Cast Metallic Core Based on the Original Prosthetic Crown

**DOI:** 10.1155/2019/6936573

**Published:** 2019-06-23

**Authors:** Eliana de Souza Bastos Mazuqueli Pereira, Fernando Accetturi, Rachel Gomes Eleutério, Daniela Vieira Buchaim, Rogério Leone Buchaim, Juliana Trindade Clemente-Napimoga

**Affiliations:** ^1^School of Dentistry, University of Marília (UNIMAR), Marília 17525-902, Brazil; ^2^Private Dental Clinic, Fernando Accetturi Implants and Aesthetic Dentistry, Marília 17525-170, Brazil; ^3^Medical School, University Center of Adamantina (UniFAI), Adamantina 17800-000, Brazil; ^4^Bauru School of Dentistry, University of São Paulo (FOB/USP), Bauru 17012-901, Brazil; ^5^School of Dentistry, São Leopoldo Mandic (SLMANDIC), Campinas 13045-755, Brazil

## Abstract

The dental crown can be restored using the root in clinical situations where the dental remnant allows adequate anchorage by the use of an intraradicular retainer. After endodontic treatment, reconstruction of the dental anatomy depends on correct planning and the type of restoration to be used. This requires reestablishment of form and function with creation of anchoring features, avoiding detachment and favoring the distribution of forces, thus preventing fracture of the remnant due to functional and parafunctional forces applied on the tooth. This paper reports the clinical case of a patient who sought dental care for a full metal-ceramic crown with a cast metallic core with reduced length that had been detached from tooth 24. After clinical and radiographic examination, root integrity was verified. The patient was offered reconstruction with a cast metallic core of satisfactory length, providing adequate retention and support, with reutilization of the original prosthetic crown, serving as a reverse template of the coronal portion of this new core, providing reduction in costs and operational time.

## 1. Introduction

Intraradicular posts and cores were originally designed to provide retention and mechanical resistance to the coronal restoration when the remaining dental structure is inadequate [1, 2]. The ability of the core to undergo stress, ease of placement and removal, compatibility with other restorative materials, and the health of supporting tissues are important factors that must be considered when their placement is required [3]. Endodontically treated teeth usually require core restorations for retention due to structural defects resulting from caries and extensive preparation for access, with significant decrease in their resistance due to the reduction of dentin moisture and compromise of strengthening structures such as marginal ridges, enamel bridge, and roof of the pulp chamber [4–6].

The dental restoration should be planned to protect the remaining structure from fractures and replace the lost dental structure and should ideally be done immediately after endodontic treatment [7–9]. The use of intraradicular retainers is indicated for esthetic and functional rehabilitation in endodontically treated teeth that have lost 50% or more of their coronal structure [10–12], requiring the use of prefabricated posts or cast metallic cores. The success of reconstruction is more dependent on the remaining dental structure and its implantation than on the choice of the intraradicular retention system.

The clinical decision may be complicated due to factors that contribute to additional weakening of these teeth associated with the compromised root canal. Despite the constant criticism, cast metallic cores have been used for decades, since they are indicated for ordinary cases, where the extent of tooth structure loss is significant and maximum retention is required. They are also very versatile, allow better adaptation to the root canal despite differences in root configurations and angulations, and present satisfactory performance in many long-term clinical studies [13–15].

Ideally, the length of cast metallic cores should be two thirds of the total length of the dental remnant [16, 17], or when the tooth being restored presents bone loss, it must be equivalent to at least half of its bone support. In addition, a minimum amount of 4 mm of obturation material should be left in the apical root portion to ensure adequate sealing of this region [17]. However, this conventional restoration method has biological and mechanical disadvantages, such as excessive tooth structure reduction, retention loss, radical fractures, and high modulus of elasticity, which induce stress concentrations at the root apex [18–21]. Thus, this case report aims at presenting the clinical reuse of an original prosthetic crown serving as a reverse template of the coronal portion of a new cast metallic core.

## 2. Case Presentation

**Figure 1:**
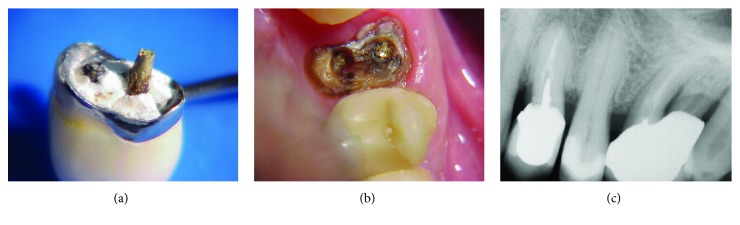
(a) Initial aspect of the metal-ceramic crown with a fractured cast core; (b) aspect of the fractured dental remnant of the cast core inside the root canal, which was later removed; (c) initial radiography.

A 43-year-old male patient attended a dental clinic with the prosthetic crown of tooth 24 in hand, with the remaining fractured root core, part of which was inside the root canal (Figures 1(a) and 1(b)).

Clinical and radiographic examination revealed the absence of a root fracture, which might preclude maintenance of the tooth (Figure 1(c)). It was also observed that the root canal had not been submitted to removal of sealing material up to the adequate length of 2/3 of the dental remnant to the root apex [16, 17]; the cast metallic core was short, which impaired the intraradicular retention.

During clinical examination, it was observed that the dental remnant presented satisfactory conditions for a new rehabilitation with placement of an intraradicular core and a total prosthetic crown. It was proposed to remove the portion of the cast core that was inside the root canal for later accomplishment of a new intraradicular cast core, using the existing metal-ceramic crown as a reverse template for the coronal portion of this future core.

This alternative was possible because there was no need for additional preparation (wear) of the dental remnant at the cervical level, which would impair the adaptation and reuse of the original prosthetic crown. Initially, root canal preparation (buccal and palatal) was performed by instrumentation with Gates Glidden drills at the appropriate length (2/3 of the dental remnant in the largest (palatal root canal), 1/2 of the dental remnant in the other canal (buccal)) [16, 17].

**Figure 2:**
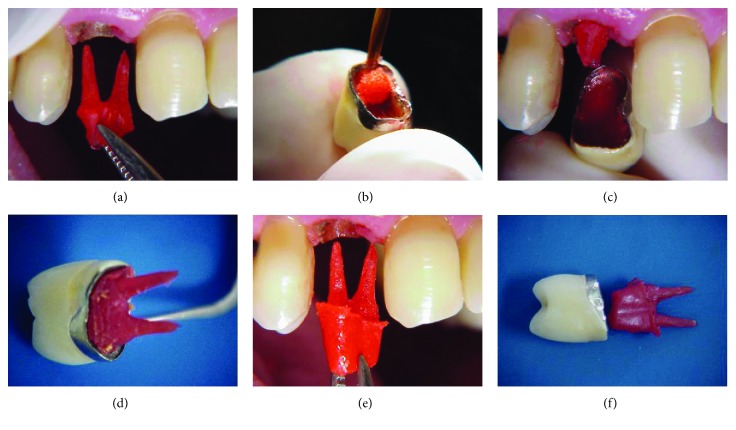
(a) Molding of the root canals with acrylic resin to make a new cast metallic core; (b) initiation of filling of the inner portion of the metal-ceramic crown with acrylic resin; (c) filling of the inner portion of the metal-ceramic crown with acrylic resin and placement on the coronal remnant of the core molded in resin; (d) fitting of the prosthetic crown on the dental remnant after placement of resin in the inner portion of the crown; (e, f) finished acrylic resin core.

Then, the prosthetic crown was internally ground with carbide drills to allow coronal adaptation of the new cast core. For that purpose, the prepared root canals were previously isolated with petroleum jelly and filled with fluid acrylic resin, supported by prefabricated resin rods (Pincanal®) in each canal. Polymerization of the acrylic resin was properly controlled to avoid adhesion of the resin pin inside the root canal (Figure 2(a)).

For preparation of the coronal portion of the core, the prosthetic crown was fitted on the coronal remnant and was internally isolated with petroleum jelly, filled with acrylic resin, and repositioned on the dental remnant with the core in place, thus serving as a reverse template for reconstruction of the coronal portion of this new core (Figures 2(b)–2(d)).

After completion of polymerization, the finished resin core was sent to the prosthesis laboratory for alloy casting (Figures 2(e) and 2(f)).

**Figure 3:**
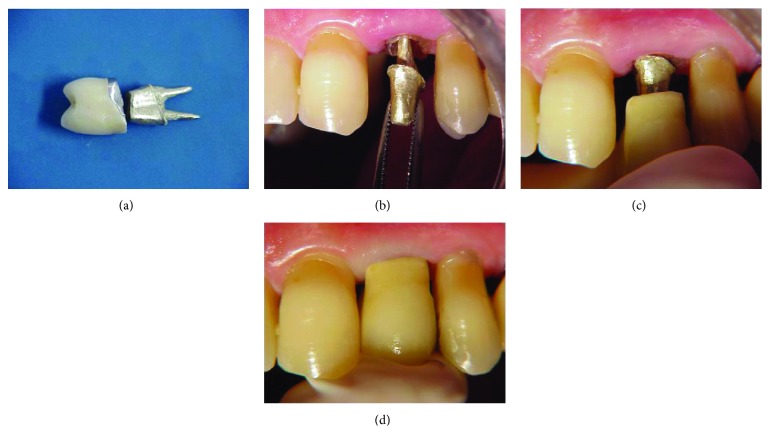
(a) Finished new cast metallic core; (b) fitting and adaptation of the cast metallic core on the dental remnant; (c) verification of adaptation of the original prosthetic crown on the new cast metallic core; (d) verification of adaptation of the original prosthetic crown on the new cast metallic core.

This new core was properly adjusted to the remaining root, and the adaptation of the metal-ceramic crown on it was also verified (Figures 3(a)–3(d)).

**Figure 4:**
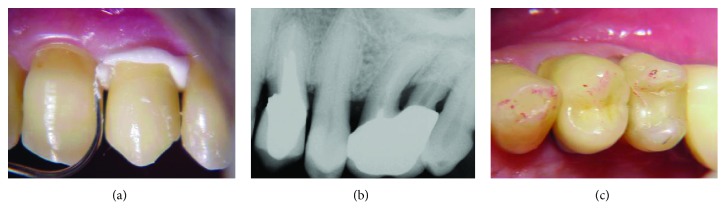
(a) Definitive cementation of prosthetic parts (core and crown); (b) final radiography; (c) verification and adjustments of occlusal contacts.

Finally, both pieces (core and crown) were cemented with definitive zinc phosphate cement (Figures 4(a) and 4(b)), and the occlusal contacts were adjusted (Figure 4(c)).

## 3. Discussion

In prosthesis and oral rehabilitation, correct reconstruction of the dental anatomy provides greater masticatory effectiveness, being a constant search of dental professionals [22]. Newman et al. stated that the need for placement of an intraradicular core is determined by two factors: the amount of remaining dentin to retain the core and the internal anatomy of the root; therefore, the professional should have the necessary knowledge to decide the best option to restore an endodontically treated tooth [23]. In this clinical case report, an alternative of reusing a metal-ceramic crown was demonstrated by performing an intraradicular reverse-cast core. This is clinically feasible when the dental remnant supports the masticatory forces applied on the tooth.

With the variety of options available to restore a tooth with great coronal destruction, it is increasingly necessary to know the main intraradicular retainer systems, so they can be adequately indicated for each specific clinical situation. It is known that the cast metallic cores and titanium posts exhibit high modulus of elasticity and high flexural strength, which allow the core to withstand large amounts of stress before it flexes and transmits the load to the root of the restored tooth, providing great resistance to this core system [23]. The cast metallic cores, together with prefabricated metallic posts, represent a good prosthetic option when the teeth to be restored are weakened for any reason (wide root canals, lack of remnants); however, the protocol for fabrication and placement of these intraradicular cores must be respected to allow treatment predictability [24].

Removal of intraradicular cores is a safe procedure and should be indicated in most situations rather than endodontic surgery or dental extraction. When the need for removal and exchange of a cast intraradicular core is required, utilization of the prosthetic crown as a template for the resin coronal portion of the new core is a viable, less expensive, and more convenient option for dental reconstruction, taking advantage of the same definitive original crown for final restoration [25, 26].

As shown in this case report, the correct observation of criteria for its indication allows utilization of the prosthetic crown previously placed in the oral cavity. Reverse casting of the intraradicular metallic core leads to resolution in shorter clinical time with reduction in the number of sessions in which the patient should be treated, in addition to a lower operating cost.
